# Editorial: Insights in pediatric endocrinology: 2024

**DOI:** 10.3389/fendo.2025.1679695

**Published:** 2025-08-28

**Authors:** Madhusmita Misra, Sally Radovick

**Affiliations:** ^1^ School of Medicine, University of Virginia, Charlottesville, VA, United States; ^2^ The University of Arizona, Tucson, AZ, United States

**Keywords:** puberty, aromatase inhibitor therapy, Prader Willi syndrome, X-linked hypophosphatemia, thyroid nodules, type 1 diabetes, metabolic syndrome associated steatotic liver disease, Rett syndrome

This Research Topic included three papers on puberty related issues. A review by Bangalore Krishna and Garibaldi discusses assays currently used for assessment of pubertal hormones and the limitations of these assays. The review emphasizes the importance of using highly sensitive assays when relying on a single early morning basal luteinizing hormone (LH) level to diagnose central precocious puberty (CPP), and the importance of confirming this with a repeat, early morning sample. The authors point out that while pubertal levels of basal LH are diagnostic of CPP, undetectable LH levels do not exclude onset of central puberty, and that such results may occur in 20 - 35% of girls and about 5% of boys in early puberty. They point out that high dose biotin supplements can interfere with results of gonadal steroid assays, and should be discontinued if results do not make clinical sense. Finally, they point out that girls with CPP may not always demonstrate a stimulated LH level following leuprolide administration of > 5 IU/L; however, the majority will achieve peak estradiol levels of > 50 pg/mL about 20 – 24 hours after leuprolide, consistent with pubertal activation. In contrast, boys with CPP do demonstrate peak LH levels of > 5 IU/L and 20 – 24 hour testosterone levels are less important.

Further, Li et al., report on the possible contribution of perfluorinated endocrine disrupters to the increased risk of CPP in girls noted during the COVID - 19 pandemic. Using metabolomics and enrichment analysis, they demonstrate an increase in levels of three perfluorinated compounds in girls with CPP compared to prepubertal controls and involvement of multiple pathways in the CPP process. They conclude that perfluorinated compounds may promote CPP in girls by interfering with pathways that impact the hypothalamic-pituitary-gonadal axis, and urge additional research on environmental endocrine disrupters.

Finally, Ebo et al. report on the validity and reliability of self-staging of puberty, particularly in the context of the use of this strategy during televisits, using data gathered during the COVID - 19 pandemic. A kappa value of ≥ 0.60 was used to indicate significant agreement of ratings of breast and pubic hair staging in girls, and testicular size and pubic hair staging in boys as assessed by the patient and the physician. The highest kappa values were evident in girls at the extremes of pubertal staging (stages 1 and 5), while for boys, the highest values were noted for Tanner stages 1 and 2 of puberty. Self-staging appears to work well in distinguishing between presence or absence of puberty, while being less useful in characterizing specific pubertal stages. In addition to televisits, these findings have important implications for the use of self-staging in research studies.

Zhu et al. examine associations of the inflammatory marker, high sensitivity C-reactive protein (hs-CRP), with sex steroid levels in prepubertal and pubertal children, and report inverse associations of hs-CRP with testosterone in pubertal boys and with estradiol in pubertal girls. In contrast, hs-CRP levels were positively associated with estradiol in prepubertal and pubertal boys, and with testosterone in prepubertal girls. BMI was associated positively and sex hormone binding globulin (SHBG) negatively with hs-CRP levels, consistent with higher BMI being associated with higher insulin and lower SHBG levels and greater metabolic risk. The study demonstrates that many factors regulate inflammatory and metabolic risk in youth.

Marin et al. discuss the use of MRI in pediatric endocrinology for conditions such as growth hormone deficiency, short stature and CPP, and report on the large proportion of normal scans (67%), the high prevalence of incidentalomas (17%), and that 86% of repeat scans were unnecessary based on established protocols. They recommend developing guidelines for scanning to optimize yield, reduce costs and minimize distress to patients and caregivers.

Giannopoulou et al. report on the impact of aromatase inhibitor therapy (letrozole) in a family with aromatase excess syndrome and demonstrate prevention of gynecomastia and improved adult height when letrozole therapy is initiated early, and improved physical strength and libido when this is started in adult life. Thus, aromatase inhibitors may be considered a therapeutic strategy in patients with aromatase excess syndrome.

The Research Topic includes two papers on Prader Willi syndrome. The paper by Wędrychowicz et al. explores the prevalence of central adrenal insufficiency (CAI) in children with Prader Willi syndrome using the low dose ACTH stimulation test, the glucagon stimulation test or both. Overall, only 1 of 46 children demonstrated convincing evidence of CAI, suggesting a low prevalence. The authors recommend against routine screening for CAI, and that the diagnosis should be confirmed with two tests to avoid unnecessary treatment with hydrocortisone. The second paper is a review of the condition by Madeo et al. and describes endocrine conditions in relation to specific genetic etiology of Prader Willi syndrome.

Wang et al. discuss data from a meta-analysis and systemic review of burosomab treatment in children with X-linked hypophosphatemia. The study includes data from eight cohort studies and two randomized controlled trials and concludes that burosumab has excellent therapeutic efficacy in treating this condition.

Januś et al. present a retrospective analysis of the histopathology underlying the ultrasound findings in benign, borderline, and malignant thyroid nodules in 47 children at a single tertiary thyroid center in Poland. Each type of thyroid nodule is characterized in detailed pathological terms, along with sonographic findings. The reader immediately notes the descriptive overlap in ultrasound findings among the different tumor types, leading the authors to conclude that ultrasonography is insufficient for accurate risk stratification, thereby necessitating fine-needle aspiration biopsy (FNAB) in children.

An increase in the prevalence of autoimmune diseases during the COVID - 19 pandemic has been reported in several studies, while others have not found an association. Hampering these studies was the lack of pre-pandemic control data. Hence, Herczeg et al., in a retrospective analysis, determined the prevalence of thyroid autoimmunity (TA) for the 10 years prior to the pandemic and during the pandemic in a cohort of 1,361 children and young adults with type 1 diabetes (T1D). The increase in the prevalence of anti-thyroid autoantibodies in children with T1D was detected during the pre-pandemic years but not during the COVID - 19 pandemic. Interestingly, 28.5% of children with anti-thyroid autoantibodies had clinically relevant thyroid-stimulating hormone (TSH) abnormalities (most commonly subclinical hypothyroidism) and/or were prescribed thyroid medication. They conclude that although there was a rise in the prevalence of thyroid autoimmunity among T1D children over the past decade, there was no association with the increase in the development of the disease with COVID - 19.

Type 1 diabetes is characterized by the destruction of pancreatic beta cells, which leads to insulin deficiency and significantly reduced levels of the exocrine pancreatic enzymes amylase, lipase, and trypsin. Bruggeman et al. aimed to determine whether the recently approved immunotherapies—anti-thymocyte globulin (ATG) and pegylated granulocyte colony-stimulating factor (GCSF)—resulted in changes in these three exocrine pancreatic enzymes that could serve as biomarkers to delineate response to treatment. Although the number of patients was small, there were interesting findings noted among responders to therapy (n=4-6), placebo “responders” (n=2), treated non-responders (n=16), and placebo non-responders (n=10). Responders were defined as having at least 60% of baseline area under the curve (AUC) C-peptide levels after a 2-hour mixed meal tolerance test (MMTT) at two years post-treatment.

Baseline levels of lipase and trypsin were lower, although not significantly, but improved to 115% of baseline in responders to immunotherapy six months after treatment. Non-responders and placebo subjects experienced a decline in lipase and trypsin to 80 - 90% of baseline during this time period. There were no differences in amylase levels between groups at baseline or six months after treatment. These preliminary findings suggest that lipase and trypsin may serve as biomarkers for response to immunotherapy in type 1 diabetes. As the authors note, further studies with larger participant numbers are needed to address this question.

In a review, Tas et al. explore whether metabolic dysfunction-associated steatotic liver disease (MASLD) may explain the increased risk of cardiovascular disease (CVD) in individuals with Type 1 Diabetes (T1D). The review manuscript focuses on observational studies, cohort studies, and meta-analyses that investigate the prevalence of MASLD in T1D populations and its association with CVD. Furthermore, the manuscript examines the physiological mechanisms that link MASLD and CVD, including hepatic insulin resistance, systemic inflammation, and atherogenic dyslipidemia, to assess the independent contribution of MASLD to cardiovascular risk in T1D patients. The literature suggests that chronic inflammation and atherogenic lipid profiles associated with MASLD elevate the risk of CVD and recommends routine assessments of liver dysfunction in the care of patients with T1D to mitigate the risk of cardiovascular complications.

Chimatapu et al. report retrospective data from a group of 42 adolescent males followed at a single center who tested sufficient on the initial growth hormone stimulation test but continued to present with short stature or growth failure. Interestingly, 59% tested deficient upon reevaluation and were started on rhGH therapy, exhibiting an excellent response. The adult height was reported for half of the patients treated with rhGH who reached adult height, which was comparable to results reported for those with IGHD. The authors emphasize the importance of re-evaluating children who show ongoing evidence of inadequate growth despite previously normal growth hormone stimulation testing. They recommend longitudinal monitoring and retesting for patients who continue to experience growth failure and may benefit from rhGH therapy. The authors hypothesize that the evolving growth hormone deficiency (EGHD) is due to ‘progressive decline or insufficient production of growth hormone (GH), especially during the period of pubertal development’.

In a case report of a 14-year-old male with insulinoma and primary hyperparathyroidism, a novel heterozygous mutation in MEN1 is reported and characterized. (Huang et al.) The mutation is also found in the proband’s father, who exhibited only hyperparathyroidism in adulthood, suggesting that family members may present variations in clinical phenotypes. The grandparents and the father’s siblings were unwilling to undergo genetic testing, opting only for screening of blood glucose, calcium, phosphate, and PTH, all of which were normal. No additional pathologic involvement of the pituitary gland, adrenal glands, or lungs was found in the proband. This manuscript underscores the importance of genetic testing in patients with MEN1 and their family members.

Rett syndrome (RTT) is an X-linked progressive neurodevelopmental disorder primarily affecting girls and is the second most common cause of genetic intellectual disability. RTT results in neurological regression between 6 and 18 months of age and is associated with varying degrees of neurological impairment. Recent data indicate that the endocrine system is frequently involved in RTT patients, including disorders of growth, bone health, the thyroid, pubertal onset, and weight abnormalities. However, systematic data on endocrinopathies in patients with RTT are limited. Pepe et al.’s systematic review manuscript aims to analyze the prevalence and types of endocrine comorbidities in the RTT population to facilitate early diagnosis and appropriate endocrinological management. Out of the 1090 studies screened, 22 met the inclusion criteria. The main endocrinopathies reported were malnutrition, bone abnormalities, and delayed puberty onset. The authors conclude that endocrinopathies are not uncommon in RTT patients and recommend screening and monitoring for endocrinopathies.

Digital health technologies are becoming an integral part of enhancing patient care and the management of chronic conditions. These technological advances can lead to increased adherence, a cost-effective healthcare system, and improved medication self-management. To assess the willingness of 22 healthcare providers to integrate the connected rhGH injection pen into their clinical practice, participatory workshops were conducted in Rome, Italy, and Seoul, Korea—two diverse healthcare ecosystems. (Rivera Romero et al.) This qualitative study explored current attitudes toward the digitalization of rhGH therapy through panel discussions, analyzed healthcare providers’ perceptions regarding the potential acceptance of the connected device compared to other non-connected alternatives (e.g., pen and paper adherence diaries), and assessed factors affecting their intent to use and integrate digital health solutions that support rhGH therapy in clinical practice. The authors conclude that understanding the nuances of these perspectives is essential for developing strategies to address the challenges and capitalize on the opportunities presented by the ongoing digital transformation in healthcare. Although healthcare providers recognize the potential of digital health solutions to enhance patient engagement and, consequently, clinical outcomes, the participatory workshops highlighted several aspects of how this digital transformation is influencing treatment options and the necessity for digital literacy for successful implementation. (Rivera Romero et al.).

Finally, a manuscript by Zucchini et al. in the Study Group on Diabetes of the Italian Society of Pediatric Endocrinology and Diabetology (ISPED) is based on a systematic review of available scientific evidence and a Delphi consensus methodology, aiming to provide evidence-based recommendations for recognizing, risk stratifying, treating, and managing patients with hypoglycemia. The objective of these recommendations is to improve the timely recognition and prevention of hypoglycemic episodes and to apply the correct treatment, especially for patients using continuous glucose monitoring (CGM) or advanced hybrid closed-loop systems. Practical flow charts are included to aid clinical decision-making, which will be very helpful for clinicians, especially when caring for patients using CGM and other advanced technologies. Importantly, the authors explore the concept of ‘fear of hypoglycemia’ (FoH), nasal glucagon use, and educational support to fully address the needs of the Italian community.

